# Fueling the fire: emerging role of the hexosamine biosynthetic pathway in cancer

**DOI:** 10.1186/s12915-019-0671-3

**Published:** 2019-07-04

**Authors:** Neha M. Akella, Lorela Ciraku, Mauricio J. Reginato

**Affiliations:** Department of Biochemistry and Molecular Biology, Drexel University College of Medicine, Philadelphia, PA 19102 USA

**Keywords:** Hexosamine biosynthetic pathway, Glycosylation, UDP-GlcNAc, O-GlcNAcylation, O-GlcNAc transferase, Cancer, Metabolism

## Abstract

Altered metabolism and deregulated cellular energetics are now considered a hallmark of all cancers. Glucose, glutamine, fatty acids, and amino acids are the primary drivers of tumor growth and act as substrates for the hexosamine biosynthetic pathway (HBP). The HBP culminates in the production of an amino sugar uridine diphosphate N-acetylglucosamine (UDP-GlcNAc) that, along with other charged nucleotide sugars, serves as the basis for biosynthesis of glycoproteins and other glycoconjugates. These nutrient-driven post-translational modifications are highly altered in cancer and regulate protein functions in various cancer-associated processes. In this review, we discuss recent progress in understanding the mechanistic relationship between the HBP and cancer.

## Hexosamine biosynthetic pathway

**Fig. 1:**
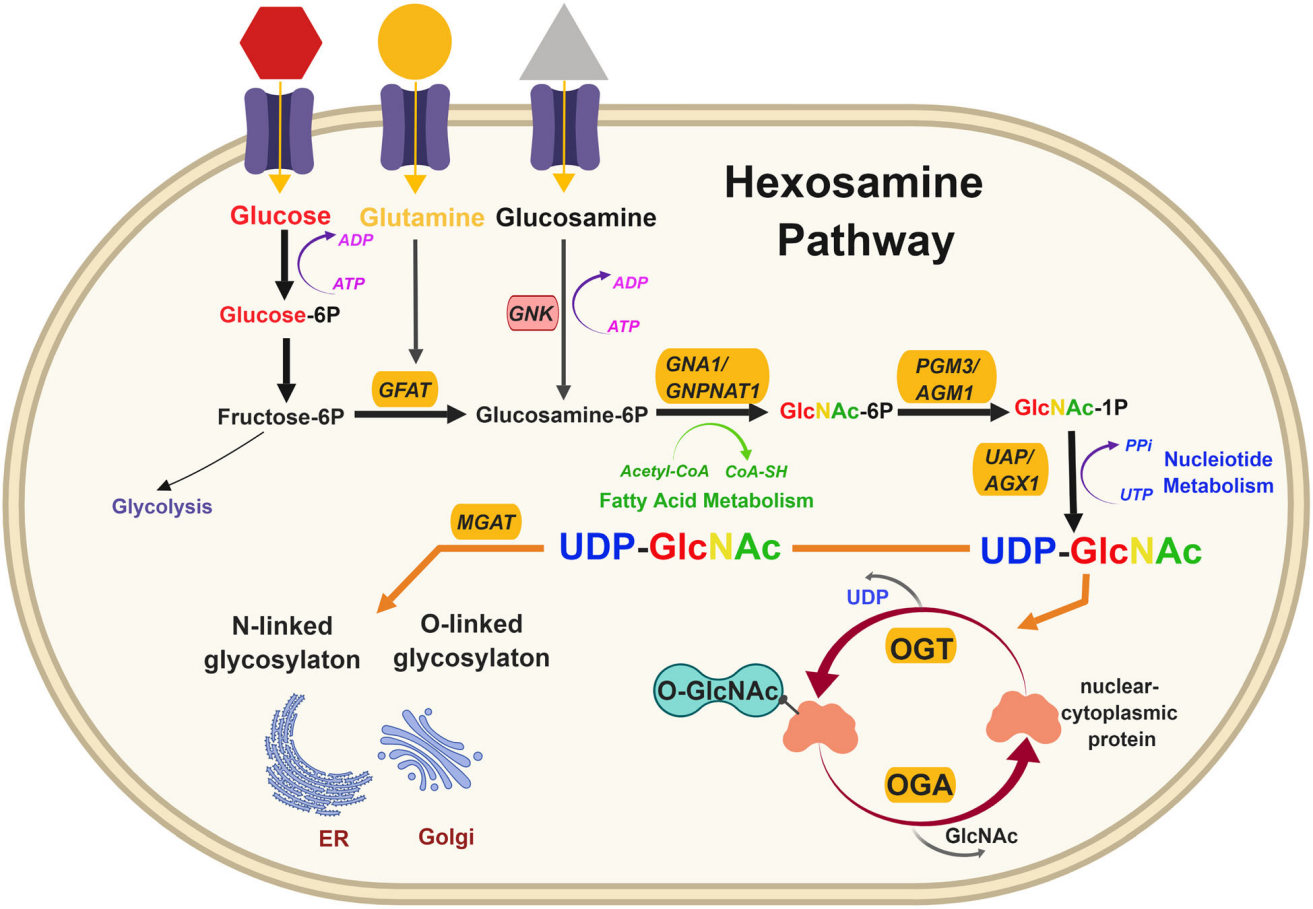
The hexosamine biosynthetic pathway. Glucose enters the cell and undergoes two-step conversion to fructose-6P (fructose-6-phosphate), after which approximately 95% of it proceeds to glycolysis and 3–5% of it is converted to glucosamine-6P (glucosamine-6-phosphate) by the enzyme GFAT (glutamine:fructose-6-phosphate amidotransferase), utilizing glutamine that enters the cell. GFAT catalyzes the first and rate-limiting step in the formation of hexosamine products and thus is a key regulator of HBP. GNA1/GNPNAT1 (glucosamine-6-phosphate N-acetyltransferase) then converts glucosamine-6P (which can also be made by glucosamine entering the cell) into GlcNAc-6P (N-acetylglucosamine-6-Phosphate), also utilizing acetyl-CoA that is made from fatty acid metabolism. This is then converted to GlcNAc-1P (N-acetylglucosamine 1-phosphate) by PGM3/AGM1 (phosphoglucomutase) and further to UDP-GlcNAc (uridine diphosphate N-acetylglucosamine) by UAP/AGX1 (UDP-N-acetylhexosamine pyrophosphorylase), utilizing UTP from the nucleotide metabolism pathway. UDP-GlcNAc is then used for N-linked and O-linked glycosylation in the ER and Golgi and for O-GlcNAc modification of nuclear and cytoplasmic proteins by OGT (O-GlcNAc transferase). OGA (O-GlcNAcase) catalyzes the removal of O-GlcNAc and adds back GlcNAc to the HBP pool for re-cycling through salvage pathway (Fig. 3)

**Fig. 2:**
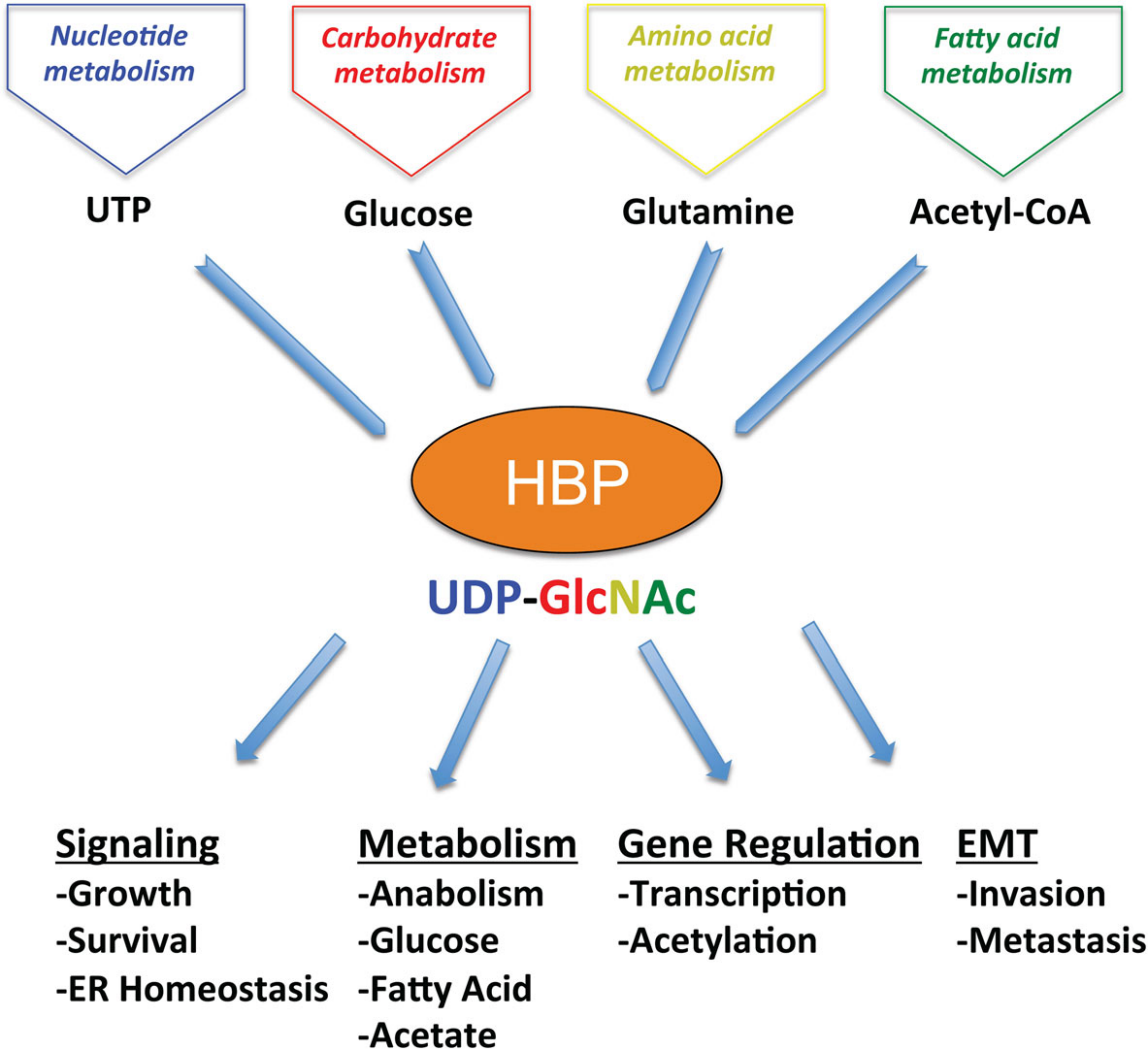
The HBP is at the center of many cancer processes. The HBP is highly dependent on the nutrient state of a cell, as is evident from its heavy dependence on dietary molecules like glucose and glutamine as well as other metabolic pathways such as nucleotide and fatty acid metabolism. The highlighted substrate UDP-GlcNAc plays a key role in orchestrating many downstream glycosylation events that in turn control proteins and processes involved in cell signaling, metabolism, gene regulation, and EMT

Nutrient sensing plays a major part in maintaining cellular homeostasis and regulating metabolic processes. The hexosamine biosynthetic pathway (HBP) and its end product uridine diphosphate N-acetyl glucosamine (UDP-GlcNAc)  are important regulators of cell signaling that favor tumor promotion. Alterations in nutrient uptake homeostasis affect cellular energetics inducing cellular stress [1]. Cell growth is primarily supported by growth factor-driven glucose and glutamine intake, which form building blocks for biosynthesis. Cells under aerobic conditions utilize oxidative phosphorylation in mitochondria to sustain energy demands. Otto Warburg noticed that cancer cells utilize far more glucose than normal cells and reprogram their metabolism largely to glycolysis even in oxygen-rich conditions [2]. This switch, termed the “Warburg effect”, funnels glycolytic intermediates into pathways that produce nucleosides, amino acids, macromolecules, and organelles required for rapid cell proliferation [3]. Unlike normal cells, cancer cells reprogram cellular energetics as a result of oncogenic transformations [4]. The hexosamine biosynthetic pathway utilizes up to 2–5% of glucose that enters a non-cancer cell and along with glutamine, acetyl-coenzyme A (Ac-CoA) and uridine-5′-triphosphate (UTP) are used to produce the amino sugar UDP-GlcNAc [5]. The HBP and glycolysis share the first two steps and diverge at fructose-6-phosphate (F6P) (Fig. 1). Glutamine fructose-6-phosphate amidotransferase (GFAT) converts F6P and glutamine to glucosamine-6-phosphate and glutamate in the rate-limiting step of HBP [6]. Glucosamine entering the cell is also converted to glucosamine-6-phosphate using GNK (GlcNAc kinase). In the next step, the enzyme glucosamine-phosphate N-acetyltransferase (GNPNAT) catalyzes Ac-CoA and glucosamine-6-phosphate to generate N-acetylglucosamine-6-phosphate (GlcNAc-6P) and CoA. This is followed by GlcNAc phosphomutase (PGM3/AGM1)-mediated isomerization into GlcNAc-1-phosphate (GlcNAc-1-P). Finally, UTP and GlcNAc-1Pz produce UDP-GlcNAc through UDP-N-acetylglucosamine pyrophosphorylase (UAP1/AGX1) enzyme [6, 7]. Since the HBP utilizes major macromolecules such as nucleotides, amino acids, carbohydrates, and lipids to produce UDP-GlcNAc, cells may use it as a ‘sensor’ of energy availability that influences a large number of functional targets that contribute to cancer phenotypes (Fig. 2).

**Fig. 3:**
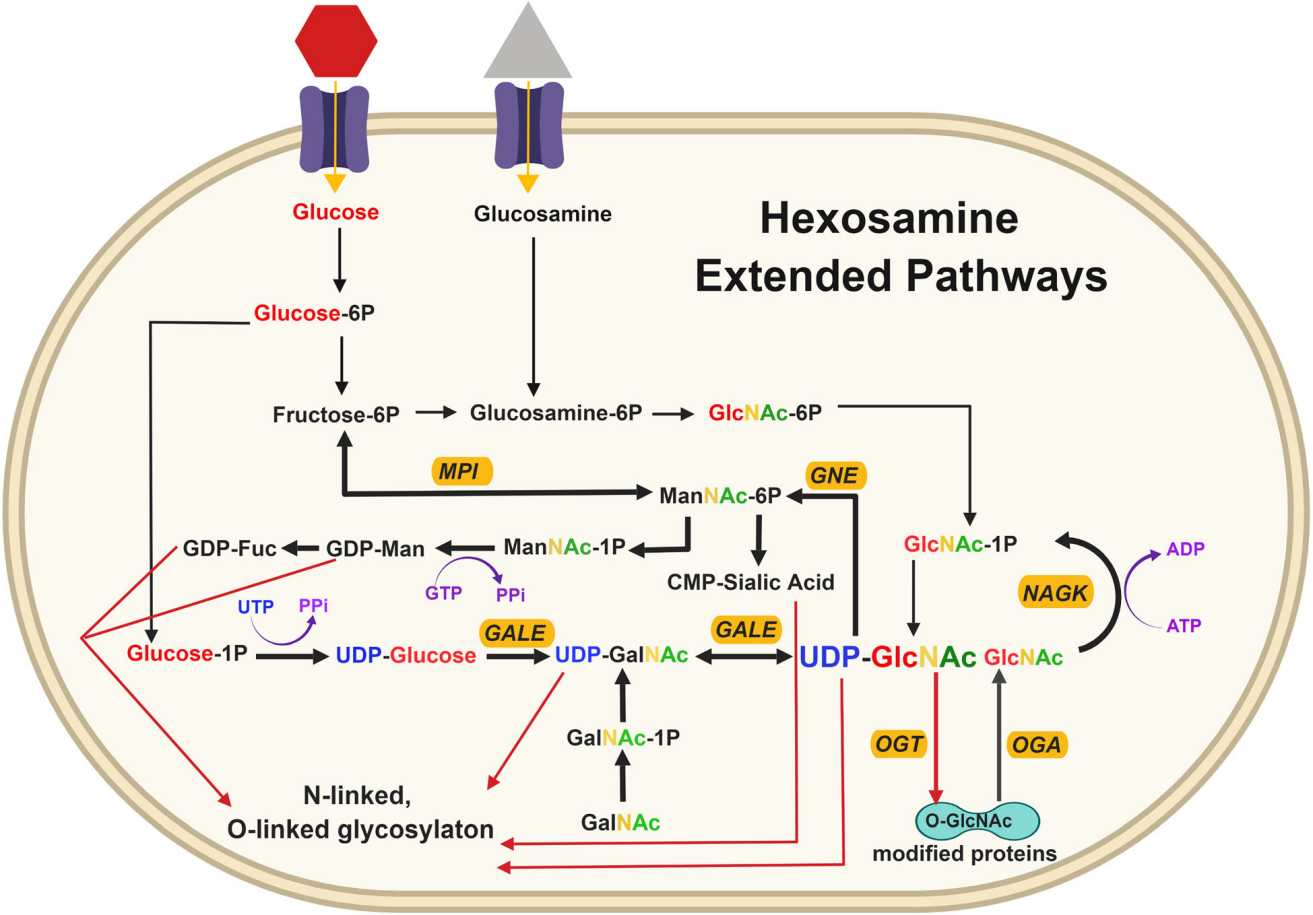
Hexosamine extended and salvage pathways. The GlcNAc salvage pathway utilizes GlcNAc via NAGK (N-acetylglucosamine kinase) to feed directly into GlcNAc-1P and produce UDP-GlcNAc . UDP-GlcNAc and UDP-GalNAc can be interconverted using GALE (UDP-glucose 4-epimerase/UDP-galactose 4-epimerase). GALE also converts UDP-glucose that comes from a three-step conversion from glucose, making more UDP-GlcNAc and UDP-GalNAc, which are both used for glycosylation in the ER and Golgi. UDP-GlcNAc can make ManNAc-6P through GNE (UDP-GlcNAc 2-epimerase/ManNAc kinase) and produce CMP-sialic acid that is utilized by the Golgi for sialylated glycoconjugation. Fructose-6P also interconverts to ManNac-6P through MPI (mannose phosphate isomerase) to produce GDP-Man (GDP-mannose) and GDP-Fuc (GDP-fucose) that are then used for glycosylation

**Fig. 4:**
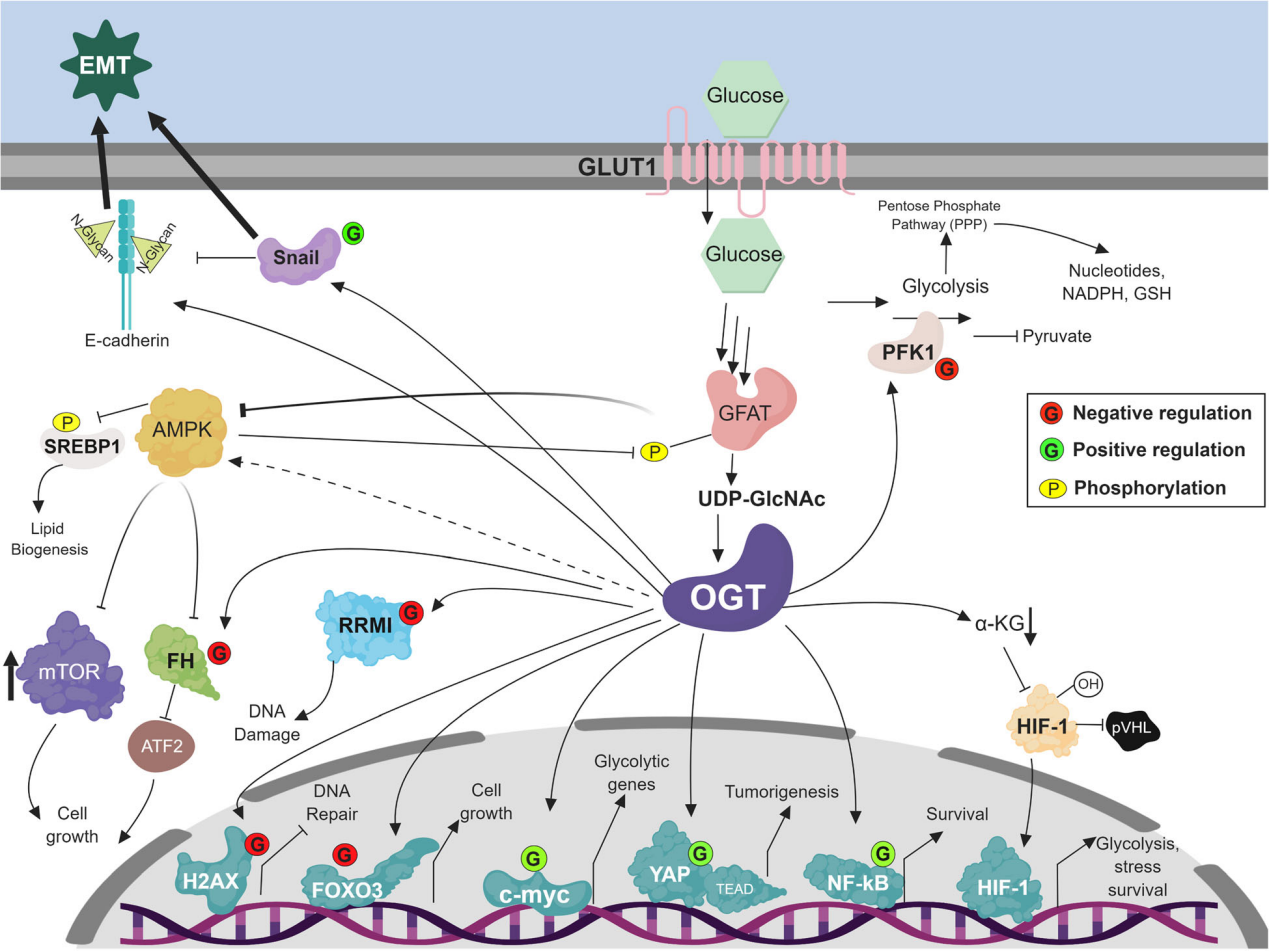
The HBP regulates multiple proteins in cancer cells via OGT. Increased glucose uptake increases HBP flux, leading to elevated UDP-GlcNAc levels and increased O-GlcNAcylation via enzymatic activity of O-GlcNAc transferase (OGT) that can positively (*green*) or negatively (*red*) regulate protein function. Increased HBP flux reduces AMPK activity and its phosphorylation of SREBP1, thus regulating lipid biogenesis. AMPK can phosphorylate GFAT and reduce HBP flux (in normal cells). O-GlcNAc modifications of transcription factors c-myc, YAP, and NF-kB result in their activation, which promotes tumorigenesis by activation of glycolytic, fatty acid synthesis, and stress survival genes while blocking expression of apoptotic genes. Elevated O-GlcNAcylation disrupts the interaction between HIF-1and von Hippel-Lindau protein (pVHL), resulting in activation of HIF-1, which upregulates GLUT1 levels and glycolytic enzymes, and increases stress survival. SNAIL O-GlcNAc modification leads to reduced levels of E-cadherin, which can be N-glycosylated upon elevated UDP-GlcNAc levels promoting EMT activation and invasive properties. The addition of a GlcNAc (G) moiety inhibits PFK1 activity, increasing flux into the PPP. Fumarase (FH) interaction with ATF2 is blocked upon its O-GlcNAc modification, resulting in failure to activate cell arrest. O-GlcNAcylation of FOXO3 and H2AX can block their function and contribute to cell growth and block DNA repair, respectively. O-GlcNAcylation of RRMI can destabilize the ribonucleotide reductase complex and cause replication stress and DNA damage

UDP-GlcNAc is required for both O-GlcNAcylation, which is a single sugar conjugation, catalyzed by O-GlcNAc transferase (OGT) in the cytoplasm, nucleus, and mitochondria [8], and O- and N-linked glycosylation of proteins occurring in the endoplasmic reticulum (ER) and the Golgi apparatus [9]. N-linked glycosylation takes place co-translationally in the ER and further N-glycan branching is added in the Golgi by four N-acetylglucosaminyltransferases (MGAT) on cell surface glycoconjugate proteins [7] (Fig. 1). UDP-GlcNAc can also be synthesized in a salvage pathway (Fig. 3) through phosphorylation of the GlcNAc molecule, a by-product of lysosomal degradation of glycoconjugates, by GlcNAc kinase (NAGK), thus bypassing GFAT [10]. GALE (UDP-glucose 4-epimerase/UDP-galactose 4-epimerase) creates another route to generate UDP-GlcNAc through interconversion of UDP-GalNAc or through UDP-glucose [11]. UDP-GlcNAc and F6P are converted to ManNAc-6-phosphate through GNE (UDP-GlcNAc 2-epimerase/ManNAc kinase) and MPI (Mannose phosphate isomerase), respectively, which goes on to further produce glycoconjugates [6, 10, 12] as described in an extended version of HBP in Fig. 3 that highlights intermediate steps not shown in Fig. 1. UDP-GlcNAc is used as a substrate to covalently modify serine (Ser) and threonine (Thr) residues of nuclear and cytoplasmic proteins solely via OGT, whereas O-GlcNAcase (OGA) is the enzyme responsible for the removal of this reversible sugar modification. O-GlcNAc modifies a wide variety of proteins, including metabolic enzymes, transcription factors, and signaling molecules (Fig. 4) [13, 14]. The extent of protein O-GlcNAcylation can also be regulated by UDP-GlcNAc localization and transport into different compartments and organelles. The nucleus and cytoplasmic levels of UPD-GlcNAc are affected by membrane permeability [14] while nucleotide sugar transporters can actively transport UDP-GlcNAc into cellular organelles such as ER and Golgi [15] as well as mitochondria [16]. In this review, we will highlight the latest discoveries into understanding the mechanistic relationship between the HBP and regulation of cancer-associated phenotypes.

## HBP and cancer

Cancer cells upregulate HBP flux and UDP-GlcNAc levels through increased glucose and glutamine uptake as well as in response to oncogenic-associated signals such as Ras [17], mammalian target of rapamycin complex 2 (mTORC2) [18, 19], and transforming growth factor beta 1 (TGF-β) [20]. Both N-linked and O-linked glycosylation can be regulated by the HBP through nutrient sensing that links to downstream cellular signaling [1, 13, 14]. An increase or depletion of extracellular glucose and glutamine levels correlates with a respective increase or decrease in UDP-GlcNAc levels in colon cancer cells [21]. Other cancers also show changes in UDP-GlcNAc levels under glucose deprivation, including cervical and pancreatic [22], hepatocellular carcinoma [23], breast cancer and pancreatic cancer cells [24], and large B-cell lymphoma [25]. In prostate cancer, GNPNAT1 and UAP1 are found to be highly expressed at the RNA and protein levels and high UDP-GlcNAc levels correlate with increased UAP1 protein levels in prostate cancer cells [26]. Targeting UAP1 in prostate cancer cells reduced UDP-GlcNAc levels and blocks anchorage-independent growth [26]. A recent study using integrative analysis of gene expression and metabolic data sets also identified alterations in the hexosamine biosynthetic pathway in prostate cancer. Compared to benign tissue, prostate cancers contained elevated levels of GNPNAT1 and UAP1 transcripts, which was consistent with increased activity of HBP in matched tumor–benign pairs as detected when levels of UDP-GlcNAc were measured [27]. Paradoxically, castration-resistant prostate cancers were found to have decreased HBP metabolites and GNPNAT1 expression, suggesting metabolic re-wiring may occur during prostate cancer progression. Nevertheless, consistent with increased UDP-GlcNAc levels in cancer cells, nearly all cancer cells examined, including from prostate [28, 29], breast [30–32], lung [33], colon [33], liver [34], endometrial [35], cervical [36], and pancreatic [37] cancer, also contain increased O-GlcNAcylation. Since many of these cancers also had increased OGT RNA and protein levels, it is not clear whether elevated O-GlcNAcylation is due to increased UDP-GlcNAc substrate availability, increased OGT levels, or both. In addition, HBP enzymes have also been found to be elevated in cancer cells, indicating they contribute to increased UDP-GlcNAc levels. For example, GFAT overexpression in colon cancer plays a role in tumor progression and metastasis as its pharmacological and genetic inhibition led to reduction of tumor size, growth, and metastasis through reduction of O-GlcNAc levels, as well as decreased expression of N-glycans [21].

HBP activity may also be increased in cancer cells by tumor microenvironment components. A recent study by Halama et al. [38] showed upregulation of HBP metabolites upon co-culturing of ovarian or colon cancer cells with endothelial cells, demonstrating a metabolic alteration only at the carbohydrate level, where the metabolites can be utilized for glycosylation or hyaluronan synthesis. Interestingly, there were no changes in glucose, lactate, or tricarboxylic acid (TCA) cycle metabolites, indicating that the Warburg effect is not occurring at the initial stage of co-culture, which suggests the HBP in cancer cells may also be activated by the endothelial microenvironment [38].

It is well established that both OGT and OGA RNA levels are responsive to alteration in O-GlcNAc signaling, suggesting existence of an O-GlcNAc homeostatic mechanism in normal cells [39–41]. For example, a rapid decrease in OGA protein expression occurs in murine embryonic fibroblasts when OGT is knocked out [42] while in hepatocytes OGA overexpression results in increased OGT mRNA levels [43]. Recent data suggest this O-GlcNAc homeostatic mechanism may be disrupted in cancer. In numerous human cancers, particularly in pancreatic adenocarcinoma, *OGT* and *OGA* expression levels are highly positively correlated [43]. In a *Kras*^*G12D*^ -driven mouse pancreatic adenocarcinoma cell line, ERK signaling may alter *O*-GlcNAc homeostasis by modulating OGA-mediated *Ogt* transcription [43]. Thus, cancer cells upregulate the HBP flux and enzymes intrinsically and oncogenic signaling pathways may alter O-GlcNAc homeostasis that contribute to increasing the HBP in cancer cells.

## HBP in cancer signaling

The HBP and its end product UDP-GlcNAc are important regulators of cell signaling that favor tumor promotion. Recent studies have shown cross-regulation between O-GlcNAcylation, mTOR, and adenosine monophosphate (AMP)-activated protein kinase (AMPK) pathway [44]. In breast cancer cells, increased mTOR activity is associated with elevation of total O-GlcNAcylation and increased OGT protein levels, while blocking mTOR activity with rapamycin leads to reduced O-GlcNAcylation and OGT levels [45]. Recently, a similar correlation between mTOR activity and O-GlcNAcylation has also been described in colon cancer cells [46]. Conversely, reducing OGT levels or O-GlcNAcylation in breast cancer cells leads to inhibition of mTOR activity as measured by phosphorylation of ribosomal protein S6 kinase beta-1 (p70S6K) [47], an mTOR target. O-GlcNAcylation has not been identified as a post-translational modification (PTM) on mTOR; thus, it is likely the HBP regulates mTOR indirectly via regulation of AMPK (see below), a negative regulator of mTOR activity. O-GlcNAcylation has also recently been shown to regulate the Hippo signaling pathway through direct O-GlcNAcylation of the oncogenic yes-associated protein (YAP). O-GlcNAcylation on Ser109 affects the transcriptional activity of YAP by interfering with its large tumor suppressor kinase ½ (LATS1/2) interaction, promoting tumorigenesis in pancreatic cancer cells (Fig. 4) [48].

The HBP also has critical crosstalk with the unfolded protein response (UPR) pathway. Human cancers have been found to be metabolically heterogeneous [49], consistent with the idea that cancer cells may be exposed to conditions of low or high nutritional states and are under constant metabolic stress [50]. Low nutritional states can trigger the UPR and ER stress response. For example, glucose deprivation leads to a decrease in HBP flux resulting in decreased levels of N-linked glycosylation, which is abundant in the ER and required for maintaining its function [51]. The subsequent reduction in N-glycosylation triggers the ER stress response in two ways. First, ER stress-induced activating transcription factor 4 (ATF4) results in an increase in the expression of GFAT1, the rate-limiting enzyme of HBP, thus increasing HBP flux [52]. Second, ER stress signals the activation of the UPR, which in turn leads to overexpression of X-box binding protein 1 (XBP1) and also to an elevation of HBP enzymes to compensate for reduced N-linked glycosylation as shown by Wang et al. [53]. Recent studies have found a critical link between the HBP and the ER stress response in cancer cells. Targeting OGT or reducing O-GlcNAcylation in cancer cells leads to metabolic stress and ER stress response, including protein kinase R (PKR)-like endoplasmic reticulum kinase (PERK) activation, increased phosphorylated eukaryotic translation initiation factor 2 alpha (p-eIF2α) and CCAAT/Enhancer-binding protein homologous protein (CHOP) levels and apoptosis [47]. Importantly, reversing metabolic stress by overexpression of glucose transporter 1 (GLUT1) or reversing ER stress by depleting CHOP reversed OGT-depleted cancer cell metabolic stress and apoptosis. A recent study treating pancreatic cancer cells with a known inducer of ER stress, 2-DG, revealed AMPK-mediated GFAT1 inhibition resulting in decreased N-glycoproteins and reduced cell growth [54]. These examples demonstrate regulation of the HBP under metabolic stress and a critical crosstalk with the UPR that contribute to cancer cell growth and survival. Overall, HBP participates in signaling pathways, primarily through O-GlcNAcylation, by regulating mTOR, AMPK, and Hippo signaling, as well as also being a downstream target of ER stress and UPR. Crosstalk between the HBP and these pathways can directly or indirectly affect the metabolic rewiring of the cell that favors tumorigenesis.

## The HBP in cancer metabolism

The HBP regulates the pentose phosphate pathway (PPP) and glutamine and glucose uptake, and functions as a bioenergetic and metabolic sensor, all of which are important to cancer cells. In cancer cells, O-GlcNAcylation and OGT play important roles in glucose metabolism as targeting OGT in breast [47] or prostate cancer cells [55] reduces glucose consumption and lactate production and is associated with reduced growth. In breast cancer cells, targeting OGT may reverse the Warburg effect as it decreases glycolytic metabolites and metabolites produced by the PPP while increasing tricarboxylic acid (TCA) metabolites [47]. This phenotype is associated with OGT regulation of GLUT1 as targeting OGT leads to reduced GLUT1 RNA and protein levels and OGT-mediated changes in metabolism and growth are reversed in GLUT1 overexpressing cells [47].

The HBP can also regulate the PPP. Phosphofructokinase 1 (PFK1), a PPP enzyme, is regulated by nutrient sensors, AMP, and fructose-2,6-bisphosphate (F2,6BP) as well as by phosphorylation. In addition, O-GlcNAcylation negatively affects the enzymatic activity of PFK1 as well, specifically by modification of Ser529 [56], a regulation seemingly specific to cancer cells (Fig. 4). This reduced PFK1 enzyme activity allows for glucose to enter the PPP, which increases production of nucleotides to support the metabolism of cancer cells, but also the production of reduced nicotinamide adenine dinucleotide phosphate (NADPH) and glutathione (GSH) to protect against oxidative stress and hypoxia. In turn, hypoxia increases glucose uptake [57], which results in increased UDP-GlcNAc and O-GlcNAcylation [58], thus stimulating PFK1 glycosylation in order to produce NADPH and cope with the metabolic stress of the cancer microenvironment.

Another important role of the HBP has been elucidated in coupling glutamine and glucose uptake to growth factor signals. Cells rely on growth factor signaling to take up nutrients and in the absence of glucose hematopoietic cells reduce the amount of glutamine uptake as well as the expression of interleukin 3 receptor (IL3-R), thus inhibiting cell growth. Wellen et al. [59] have shown that, upon extracellular supplementation of HBP-metabolite N-acetylglucosamine, glucose-starved cells were able to restore IL3-Rα cell surface expression and mediate uptake of glutamine, which enters the TCA cycle, allowing for energy production and cell growth [59]. Thus, the HBP can restore growth factor signaling and glutamine uptake in the absence of glucose.

Another important cellular process that may be affected by the HBP is AMPK, a critical bioenergetic sensor in cancer cells. Under metabolic stress and low levels of ATP, AMPK responds by inhibiting cell growth signaling pathways such as mTOR while stimulating energy production through increased fatty acid oxidation [60]. AMPK can inhibit GFAT by phosphorylating it and thus decreasing the UDP-GlcNAc pool (Fig. 4) [61]. AMPK is O-GlcNAc modified in vitro by OGT at its α and ɣ subunits, leading to increased AMPK activity; however, the role of this O-GlcNAcylation has not been examined in the cancer context [62]. AMPK behaves as a sensor even in the presence of increased HBP flux. For example, under high input of HBP nutrients, AMPK activity is diminished. Conversely, under low HBP metabolites, AMPK is activated [62]. Consistent with these data, reducing O-GlcNAcylation in cancer cells genetically or pharmacologically increases AMPK activity and reduces lipogenesis associated with increased AMPK-dependent phosphorylation of master lipid regulator sterol regulatory element binding protein (SREBP1; Fig. 4) [63]. Thus, the HBP, and specifically its ultimate product, UDP-GlcNAc, can serve as sensors and regulate the major metabolic pathways activated in cancer cells, including glycolysis, glucose and glutamine uptake, the pentose phosphate pathway, and lipogenesis.

## HBP and transcription

In order for cancer cells to support increased metabolism and proliferation, regulation of genes responsible for cell growth and proliferation is necessary. This can be accomplished through transcription factors responsible for these processes. O-GlcNAcylation of c-Myc at Thr58 competes with phosphorylation by glycogen synthase kinase 3 beta (GSK3β) and thus allows for stabilization and enhancement of the transcriptional activity of c-Myc (Fig. 4) [64]. Increased stability of c-Myc can in turn activate expression of glycolytic genes or glutamine transporters that allow for glutamine uptake and upregulation of the mitochondrial metabolism that can help provide the energy required for rapid proliferation [65]. In addition, a feed forward loop may exist between c-Myc and OGT as OGT protein levels are regulated in breast cancer cells by c-Myc. Myc stabilizes OGT protein levels via expression of the c-Myc transcriptional target heat shock protein 90 alpha (HSP90A) [45].

The guardian of the genome, p53, serves as a tumor suppressor and is thus mutated or silenced in multiple cancers [66]. O-GlcNAcylation plays an important role in conferring stability to p53 as the addition of GlcNAc to Ser149 prevents phosphorylation at Thr155 [67], thus blocking ubiquitin-dependent proteolysis and stabilizing p53. More recent studies have shown that both OGT and OGA overexpression stabilize wild-type but not mutant p53 in ovarian cancer cells, yet they found no evidence of direct p53 O-GlcNAcylation [68]. However, the functional role of O-GlcNAcylation in regulating the tumor suppressor function of p53 has not been directly examined. Forkhead box O3 (FOXO3) is a known tumor suppressor that represses cell-cycle progression and thus effectively represses abnormal cell division [69]. In pancreatic cancer cells, FOXO3 is highly O-GlcNAc modified on S284 and this modification blocks FOXO3 function, leading to subsequent cancer cell growth (Fig. 4) [70]. O-GlcNAcylation can also directly regulate the activity of the nuclear factor kappa-light-chain-enhancer of activated B cells (NF-κB) transcription factor. O-GlcNAcylation of NF-κB on Thr352 and Th322 in pancreatic cancer cells [37] stabilizes and increases its activity, as it prevents the binding of nuclear factor of kappa light polypeptide gene enhancer in B-cells inhibitor, alpha (IκBα), an inhibitor of NF-κB, thus allowing NF-κB to enter the nucleus and block apoptosis (Fig. 4).

O-GlcNAc can also regulate transcription indirectly via regulation of cancer metabolism. Elevated O-GlcNAcylation in breast cancer cells decreases TCA metabolite α-ketoglutarate (α-KG), leading to reduction of hypoxia inducible factor 1 alpha (HIF-1α) hydroxylation and interaction with von Hippel-Lindau protein (pVHL). This in turn results in HIF-1α stabilization and increased expression of its transcriptional targets, including GLUT1, and plays a key role in metabolic stress survival (Fig. 4) [47]. Recent evidence demonstrates that metabolic enzymes are able to critically affect epigenetic regulation through activity-catalyzed conversion of metabolic substrates [71]. One example is fumarase (FH), which is located in both mitochondria and the cytosol and mediates the reversible hydration and dehydration of fumarate to malate in the TCA cycle in mitochondria and amino acid and fumarate metabolism in the cytoplasm. The local fumarate produced from promoter-associated FH blocks lysine-specific demethylase 2A (KDM2A) activity, resulting in histone H3 lysine 36 methylation (H3K36me2) stabilization and transcription of activating transcription factor 2 (ATF2)-targeted genes responsible for cell growth arrest in cancer cells [72]. Interestingly, upregulated OGT activity in cancer cells leads to O-GlcNAcylation on FH-Ser75, competes with AMPK-mediated phosphorylation, compromises FH–ATF2 signaling, and prevents tumor growth arrest (Fig. 4) [72].

OGT has also been shown to directly regulate epigenetics by interacting with the Ten-Eleven translocation (TET)-family dioxygenases [73, 74], which successively oxidize 5-methylcytosine in DNA and thus promote DNA methylation [75]. TET2 and TET3 can recruit OGT to the chromatin [76, 77] and promote OGT activity on histones, specifically O-GlcNAcylation of Histone 2B at Ser112 around transcriptional start sites [76] or O-GlcNAcylation of host cell factor 1 (HCF1), which is part of the SET1/COMPASS complex, a critical H3K4 methyltransferase complex [73]. O-GlcNAcylation of TETs has also been reported [78] where OGT promotes TET3 localization to the cytoplasm [79] and O-GlcNAcylation of TET1 regulates its expression in embryonic stem cells [74]. Interestingly, TET2 is a critical regulator for hematopoietic stem cell homeostasis and a tumor suppressor whose functional impairment leads to hematological malignancies [80]. Recent studies have implicated loss of TET’s function in increasing genomic instability, reducing DNA damage repair and contributing to cancer progression [81, 82]. However, it is still not clear whether TET–OGT interaction and co-regulation are related to TET’s tumor suppressor function**.** Overall these data demonstrate an important role of the HBP, specifically through O-GlcNAcylation, in controlling the activity of key transcription factors, epigenetic regulators that regulate growth, survival, and metabolism, thus fueling cancer progression.

## HBP and epithelial to mesenchymal transition

Epithelial to mesenchymal transition (EMT) is a unique, reversible, epithelial cell property that allows for the plasticity required for various cellular processes like development, wound healing, and stem cell preservation [83]. During these events, epithelial cells lose cell–cell adhesions, undergo cytoskeletal reorganization, lose expression of epithelial proteins, and gain expression of mesenchymal proteins. The final steps of the transition involve conversion of the epithelial cell into a mesenchymal cell with migratory properties [84]. Cancer cells can co-opt and reactivate EMT and it is considered to be one mechanism that allows tumor cells to escape primary sites, invade through the basement membrane, and metastasize to distant organs [85]. Induction of EMT involves extracellular signaling from the microenvironment and expression of many transcription factors, surface-glycoproteins, extracellular matrix (ECM) proteins, cytoskeletal proteins, and extracellular-signaling from the microenvironment [84, 86].

Recent studies demonstrate strong correlations between HBP and EMT [20, 86–88]. EMT induction by TGF-β can increase glucose uptake in breast [89], colon [90], and lung [20] cancer cells. Consequently, EMT can result in high UDP-GlcNAc levels that contribute to altered glycosylation patterns on glycolipids, elevated glycosyltransferases, increased O-GlcNAcylation, and special glycosylation of fibronectin in tumor cells [91]. Moreover, it is well described that tumorigenesis and metastasis are associated with elevation of sialylation, fucosylation, O-glycans and N-glycans [20]. EMT may be responsible for some of these changes as EMT is associated with increased hybrid type N-glycans and decreased bi-, tri-, and tetra-antennary complex N-glycans in bladder cancer [92]. Key proteins involved in EMT are known to be glycosylated. For example, E-cadherin and N-cadherin have multiple N-glycosylation sites that alter the protein’s localization and stability [93]. A similar regulation of EMT by N-glycosylation is observed in integrins, where specific N-glycosylation of integrins is associated with its role in cancer cell motility and mesenchymal transition [94, 95]. In addition, there is growing evidence that glycans play an important role in EMT in cancer [96]. Integrins, receptor tyrosine kinases, Wnt, Notch and Hedgehog pathway proteins, and hyaluronic acid are all known to be N-linked glycosylated and play a role in EMT [87]. Congruently, it has been shown that, upon reduction of hyper-O-GlcNAcylation in breast and liver cancers, E-cadherin expression is increased, accompanied by a decrease in vimentin, a mesenchymal marker [37, 97, 98]. Specifically, O-GlcNAcylation of E-cadherin blocks its cell surface transport, therefore favoring cell migration [99], a process juxtaposed by the O-GlcNAcylation of zinc finger protein SNAI1 (Snail), which causes reduced expression of E-cadherin, similarly leading to migration [97]. Guillaumond’s group [100] showed that hypoxic areas in pancreatic ductal adenocarcinoma (PDAC) mouse models display an EMT signature that is associated with increased glycolysis and overexpress HBP genes like GFPT1 (by 1.5-fold) and GFPT2 (by ninefold). Another group identified a “mesenchymal metabolic signature” (MMS) [101] in which key HBP enzymes (GFPT2, GALNT10, UAP1) are upregulated in mesenchymal cells, correlating HBP with EMT [26, 86]. To the contrary, reduction of GFAT was capable of inducing EMT in gastric cancers that inherently express low GFAT, suggesting the importance of maintaining a precise balance of this pathway [102].

Another indication that the HBP contributes to EMT is data showing that altering O-GlcNAc levels alone can alter EMT. In lung cancer cells, targeting OGT led to a decrease in the mesenchymal marker N-cadherin, with an increase in E-cadherin, and conferred a more epithelial morphology [20]. Conversely, targeting OGA in these same cells led to high O-GlcNAcylation in cells, increased N-cadherin levels, reduced E-cadherin levels, increased mesenchymal morphology, and increased cell motility in the presence of TGF-β. One mechanism by which OGT can directly regulate EMT is via regulation of EMT-related transcription factors. For example, Snail is phosphorylated by CK-1 and GSK-3β sequentially and targeted for nuclear export, after which it is sent to the proteasome for degradation. However, under hyperglycemic conditions, O-GlcNAc occupies the Snail phosphorylation site on Ser112, preventing degradation and thereby stabilizing its levels (Fig. 4) [84]. In addition, OGT may also regulate EMT through E-cadherin cell-surface localization. OGT also modifies p120 and β-catenin, which directly bind E-cadherin and dictate its cell surface distribution and might therefore play a role in breast cancer metastasis. [98]. Other proteins associated with EMT, including TGF-β, NF-κB, and FOXO-1, have also been shown to be O-GlcNAc modified [84]. However, a specific role of O-GlcNAcylation of these proteins in EMT has not been investigated. Taken together, these findings suggest that increased HBP flux plays an important role in regulating EMT.

## HBP and DNA damage

The connection between cancer metabolism and DNA damage is becoming increasingly clear [103]. O-GlcNAc is a well-known regulator of the cellular stress response and can directly regulate proteins involved in DNA damage and repair [104]. OGT can modify H2AX on S139 and negatively regulate DNA double-strand break-induced phosphorylation of H2AX, leading to decreased γH2AX formation on DNA damage sites (Fig. 4) [105]. A recent report shows that reducing OGT expression in breast cancer cells was associated with defects in double-stand break repair, reduced cell proliferation, and increased cell senescence in vivo [106]. Conversely, promoting O-GlcNAcylation by targeting OGA protected tumor xenografts from radiation, thus implicating O-GlcNAcylation as a key player in the DNA damage response in cancer cells and as a potential regulator of tumor radiosensitization.

A new emerging idea is that altered metabolic states may lead to replication stress and DNA damage, and contribute to cancer-causing mutations [103]. A provocative recent manuscript shows that culturing pancreatic cells under high glucose conditions leads to replication stress and increases KRAS^G12D^ mutations [107]*.* Interestingly, high glucose treatment of pancreatic cells increased UDP-GlcNAc levels, and targeting OGT with RNA interference reduced glucose-mediated replication stress and the number of KRAS^G12D^-positive pancreatic cells. Mechanistically, these authors showed that elevated O-GlcNAcylation leads to decreased dNTP pools through O-GlcNAcylation of RRM1, a subunit of the ribonucleotide reductase (RNR). O-GlcNAcylation of RRM1 at T734 destabilizes the formation of functional RNR complex and contributes to DNA damage (Fig. 4). Thus, high glucose levels can increase HBP flux that may contribute to replication stress and possibly lead to cancer initiation in pancreatic cells. This is of potential clinical relevance as diabetic patients have an increased pancreatic cancer risk [108]. Further studies are needed to test whether over-activation of the HBP can lead to mutations and cancer development and progression.

## HBP and cancer stem cells

Emerging data suggest a potential important role of the HBP in pluripotency and possible involvement in tumor initiation through regulation of cancer stem cells (CSCs). The CSC model proposes that a subset of cancer cells within a tumor constitute a distinct population of tumor-initiating cells that contain properties of self-renewal and the ability to generate both further stem cells and differentiated cells forming the bulk of the primary tumor [109, 110]. This tumor heterogeneity poses an additional challenge of varied sensitivity to therapy between tumor subpopulations, which contributes to tumor recurrence [111]. Metabolic reprogramming is a major factor during the transition of somatic cells into pluripotent stem cells and this feature manifests in the case of CSCs as well [112]. The HBP has been highlighted as having a developmental role in mouse embryonic stem cells [113]. OGT is directly linked to Yamanaka factors like octamer-binding transcription factor 4 (Oct4) and SRY (sex determining region Y) box 2 (Sox2) where it is responsible for maintaining pluripotency and self-renewal [114, 115]. These studies, along with the lethality of the OGT [116] and OGA gene knockouts in mice [117], suggest a potential important role of the HBP in pluripotency and possible involvement in tumor initiation through regulation of CSCs.

Some recent evidence linking O-GlcNAc/OGT to regulation of CSCs is beginning to emerge. A colon cancer cell study identified hypermethylation of transcription factor Myb-related protein B (MYBL1) under high O-GlcNAc conditions as contributing to tumor progression and self-renewal [118]. Furthermore, a breast cancer study links hyaluronan over-expression to increased HIF-1α production through upregulated glycolytic flux. This positive feedback loop offers a constant supply of HBP-coupled HIF-1α signaling that is required for mammosphere formation and maintenance of the CSC (CD44^H^ CD24^L^) population [119]. A similar positive correlation of CSC properties and markers is observed with elevated GFAT1 levels [89, 119–121]. Additionally, liver cancer stem cell populations, as measured by CD133 cell-surface marker, are reduced following Azaserine (a glutamine analog and GFAT1 inhibitor) treatment as well as glucose deprivation, and this effect can be rescued with GlcNAc in glucose-deprived cells [120]. In lung and colon cancer cells, IL-8 is able to enhance CSC-associated sphere formation in vitro and tumor initiation in vivo by upregulating GFAT expression, glucose uptake, Sox2 expression, and total O-GlcNAcylation in a GLUT-3-dependent manner [121]. A recent report identified O-GlcNAc modification of eIF4E in hepatocellular carcinoma on Thr168 and Thr177. OGT and eIF4E are required for sphere formation, CD133+ expression, and expression of Oct4 and Sox2. Exogenous expression of eIF4E rescues the inhibitory effect of OGT knockdown and glucose analog (2-DG) treatment. They also found that eIF4E binds to the Sox2 5′ UTR, which could enhance translation and thus contribute to CSC properties [122]. A recent study showed that the protein product of the developmental gene BMI-1 (B cell-specific Moloney murine leukemia virus integration site 1) could be stabilized through O-GlcNAc modification at S255 in prostate cancer. Microarray analysis highlighted co-regulation of the phosphatase and tensin homolog (PTEN), p53, and cyclin dependent kinase inhibitor 1A (CDKN1A) pathways by OGT and polycomb complex protein BMI-1. This study only explored the effect of BMI-1 O-GlcNAcylation on prostate cancer cell proliferation, apoptosis, and invasion, and thus it is not clear whether BMI-1 O-GlcNAcylation contributes to prostate cancer tumor-initiation cells [123]. These studies begin to shed light on the contribution of HBP, as well as O-GlcNAc modifications, to cancer cell stemness. Currently, it is not clear whether HBP regulates a general cancer stem cell pathway or cancer-specific stem cell pathways; thus, more studies are warranted to understanding molecular links between the HBP and cancer stem cell activity.

## The HBP as a target for cancer therapy

Given the role of the HBP in driving tumorigenesis and sustaining growth and survival, it is a promising pharmacological target. Glutamine analogs like azaserine (Aza) and 6-diazo-5-oxo-L-norleucine (DON) can inhibit the HBP and show anti-tumor activity in vitro [25] and in vivo [124]. One group showed that elevated O-GlcNAcylation in acute myeloid leukemia cells is responsive to DON treatment and showed it can reduce O-GlcNAcylation, and c-Myc and c-Myb levels, and ultimately lead to apoptosis. DON treatment also reduced tumor burden in mice and did not alter the hematopoietic cell population, suggesting that it may not be toxic to normal hematopoietic cells [125]. Similarly, Aza and DON treatment [59] led to decreased levels of the intracellular UDP-GlcNAc and, consistent with GFAT gene silencing, blocked tumor cell growth [21]. Another group using a diffuse large B-cell lymphoma (DLBCL) model saw reduction in cancer phenotypes following Aza treatment. DLBCL cells show increased uptake of glucose and glutamine, increased O-GlcNAc, and activation of transcription factors NF-κB (downstream of HBP) and NFATc1 (downstream of B-cell receptor). Treatment of DLBCL cells with Aza reduced O-GlcNAc levels, inhibited activation of NF-κB and NFATc1, and induced cell cycle arrest followed by apoptosis [25]. Many studies have shown that these glutamine analogs block the HBP and O-GlcNAcylation and may correlate with its anti-tumor effects. However, it is unlikely all anti-tumor effects can be directly linked to HBP inhibition as these compounds also function as purine antagonists and glutamine amidotransferase inhibitors. For example, DON has been reported to inhibit at least eight different glutamine utilizing enzymes [126, 127]. Thus, these compounds are not HBP-specific, may have off-target effects that may contribute to toxicity, and have serious limitations in targeting this pathway in cancer cells.

Other enzymes in the HBP pathway have been targeted with small molecules, including phosphoglucomutase 3 (PGM3). This enzyme converts N-acetylglucosamine-6-phosphate to N-acetylglucosamine-1-phosphate in the HBP pathway. Pharmacological inhibition of PGM3 with the small molecule FR054 negatively affected integrin β1 localization, adhesion, and migration of breast cancer cells, and reduced tumor growth in xenograft mouse models. Targeting PGM3 decreased intracellular UDP-GlcNAc, branched N-glycans, and O-GlcNAc-modified proteins, which in turn initiated ER stress and apoptosis through ROS induction [128]. Preliminary efficiency of this drug is promising, but there is still a long way to go to achieve optimal stability, potency, and safety. However, the biggest challenges with these inhibitors continue to be cell permeability, specificity, potency and toxicity. Van Aalten’s group recently tried to address the issue of potency by developing a UDP-peptide conjugate as inhibitors of OGT. They introduced a thio-propyl linker that increased the binding potency of a UDP-peptide conjugate to the hOGT peptide in the micromolar range [129]. Vocadlo’s group has been working on the compound Ac-5 s-GlcNAc, a competitive OGT (salvage pathway) inhibitor. It successfully blocks breast cancer cell growth in vitro [47, 63, 130], but one group observed it has fairly broad specificity, inhibiting other glycosyltransferases as well [131]. This compound has good permeability and is not toxic but has low aqueous solubility, making it difficult to use in mammals. Recently, a new analog to Ac-5 s-GlcNAc, 2-deoxy-2-N-hexanamide-5-thio-d-glucopyranoside (5SGlcNHex), was generated to increase its solubility in animals and it was shown to decrease in O-GlcNAc levels in a dose-dependent manner in various mouse tissues after intraperitoneal injection. This inhibition was also reversible, where O-GlcNAc levels returned to baseline after 16 h of treatment, while not altering other protein glycosylation even at a high dose of 300 mg/kg [132]. Importantly, mice only became moribund following dosing of 300 mg/kg for two days, suggesting there may a therapeutic window to reduce O-GlcNAcylation in cancers but minimize possible toxicities.

Moreover, HBP-inhibiting drugs may have more promising utility when used in combination with current anti-cancer therapeutic agents as a number of studies have shown alteration of anti-tumor effects in vitro by these agents in combination with targeting the HBP [133–135]. Nevertheless, new drugs targeting enzymes in the HBP pathway are urgently needed for testing in preclinical cancer models to determine the suitability of this pathway as a potential target for cancer therapy.

## Future directions

Elevated HBP and O-GlcNAcylation has been reported in nearly all cancers examined and can regulate many “hallmarks of cancer”, including growth, survival, metabolism, angiogenesis, and metastasis [136]. O-GlcNAcylation is required for growth in many tumors yet it is still not clear whether HBP/O-GlcNAcylation functions as a tumor promoter or plays a fundamental role in cancer initiation and maintenance. Continued work on the role of HBP/O-GlcNAc in CSCs and tumor initiation may address this question. Developing specific chemical inhibitors of HBP enzymes is critical for understanding the role of this pathway and its possible clinical utility in treating cancer. However, as is the case with many metabolic enzymes, inhibitors of the HBP pathway may also have secondary and detrimental effects on immune cells. Recent studies have shown the HBP and O-GlcNAc are highly elevated in activated T cells and targeting OGT with Ac-5SGlcNAc [137] or targeting OGT genetically is detrimental to proliferation and clonal expansion of T cells [138]. Thus further study is needed to understand the role of the HBP in immune cells in relation to cancer. Nevertheless, the HBP has emerged as a major contributor to and regulator of cancer pathways and phenotypes. Up to this point, nearly all evidence suggests that the HBP helps fuel cancer cell metabolism, growth, survival, and spread. Further research should elucidate whether the HBP plays a role in cancer initiation and maintenance, heterogeneity, and regulation of the tumor microenvironment, including immune surveillance.

## Data Availability

Not applicable.
